# Contact dermatitis associated with the Bispectral index™ sensor: a case report

**DOI:** 10.1186/s40981-020-00393-w

**Published:** 2020-10-28

**Authors:** Noriko Taguchi, Shijima Taguchi, Syoichiro Ishizuki, Hiroyuki Ito

**Affiliations:** 1 Department of Anesthesiology, Ibaraki Prefectural University of Health Sciences, 4649-2 Ami, Ami-machi, Inashiki-gun, Ibaraki, Japan; 2 Department of Dermatology, University of Tsukuba/Mito Kyodo General Hospital, Mito, Ibaraki, Japan; 3 Department of Dental Surgery, University of Tsukuba/Mito Kyodo General Hospital, Mito, Ibaraki, Japan

**Keywords:** Bispectral index sensor, Contact dermatitis, Complication

## Abstract

**Background:**

Contact dermatitis caused by electroencephalography electrodes is rare and insufficiently studied. We described a case of contact dermatitis caused by Bispectral Index (BIS) monitor electrodes.

**Case presentation:**

A 38-year-old woman underwent tooth extraction under general anesthesia with BIS monitoring. She noticed erythema on her forehead 3 days after surgery, which peaked on the fifth postoperative day. Slight pigmentation was observed at 42 days after surgery. We performed patch testing and confirmed positive reactions to the sensor and some allergens.

**Conclusions:**

Many reports have attributed contact dermatitis to an allergen present in electrocardiogram electrodes. It is important to recognize that complications similar to those caused by electrocardiogram electrodes can occur with this sensor**.**

## Background

The Bispectral Index™ (BIS) monitor is commonly used to adequately maintain the depth of general anesthesia during surgery. The sensor comprises four electrodes and is attached on the forehead. Because the BIS monitor is placed on the forehead, dermatitis, which can lead to pigmentation, can result in serious cosmetic implications for the patient. Contact dermatitis associated with this sensor is rare, and it has not been sufficiently examined. We experienced a case of contact dermatitis involving the BIS sensor and performed patch testing. This report describes the case and discusses the results of patch testing.

## Case presentation

A 38-year-old female patient with American Society of Anesthesiologists (ASA) physical status 1 underwent tooth extraction under general anesthesia. She had no skin disease or history of allergy preoperatively, and she was receiving no medications. In the operating room, electrocardiogram (ECG) electrodes (Vitorode L, Nihon Kohden, Tokyo, Japan) and other standard noninvasive ASA monitors were attached. Immediately before induction, an electrode for the BIS monitor (BIS QUATRO Brain Monitoring Sensor, Medtronic, Dublin, Ireland) was attached in a routine manner after alcohol skin preparation as recommended by the manufacturer. Propofol and remifentanil were used for induction, and rocuronium was used for nasal intubation. Anesthesia was maintained with desflurane and oxygen to ensure a BIS of 40–60. We used eye patches (Nichiban, Tokyo, Japan) for eye protection during surgery. The durations of anesthesia and surgery were 85 and 43 min, respectively. During surgery, hydrocortisone 100 mg and acetaminophen 1000 mg were administered to prevent laryngeal edema and analgesia, separately. After surgery, the eye patches and BIS sensor were removed in the operating room. No abnormal skin reaction was noted in the region surrounding the BIS sensors, ECG electrodes, and eye patches.

**Fig. 1:**
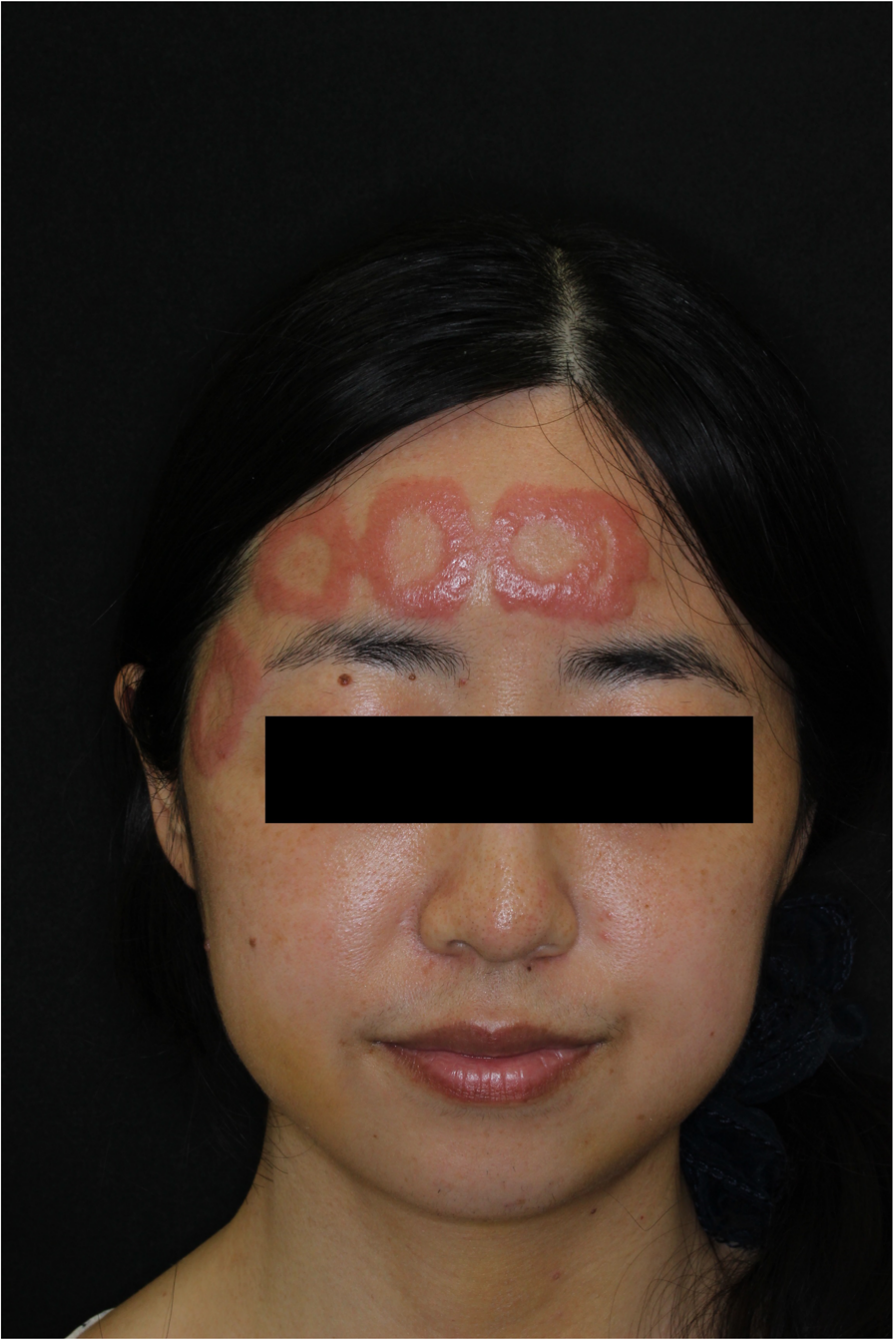
Itchy and painful erythematous lesions on contact sites of the adhesive component of the Bispectral Index sensor 3 days after surgery

The patient had no apparent skin reaction on postoperative day 1. The anesthesiologist made a postoperative round the morning after surgery and found no complications. Subsequently, the patient was discharged from the hospital. Three days after surgery, she felt itching sensations and burning pain and noticed erythema on her forehead. She visited a dermatologist 5 days after surgery, and the anesthesiologist identified dermatitis (Fig. 1). The patient was administered topical steroids and antihistamines. Erythema peaked on the fifth postoperative day and improved gradually thereafter.

**Fig. 2:**
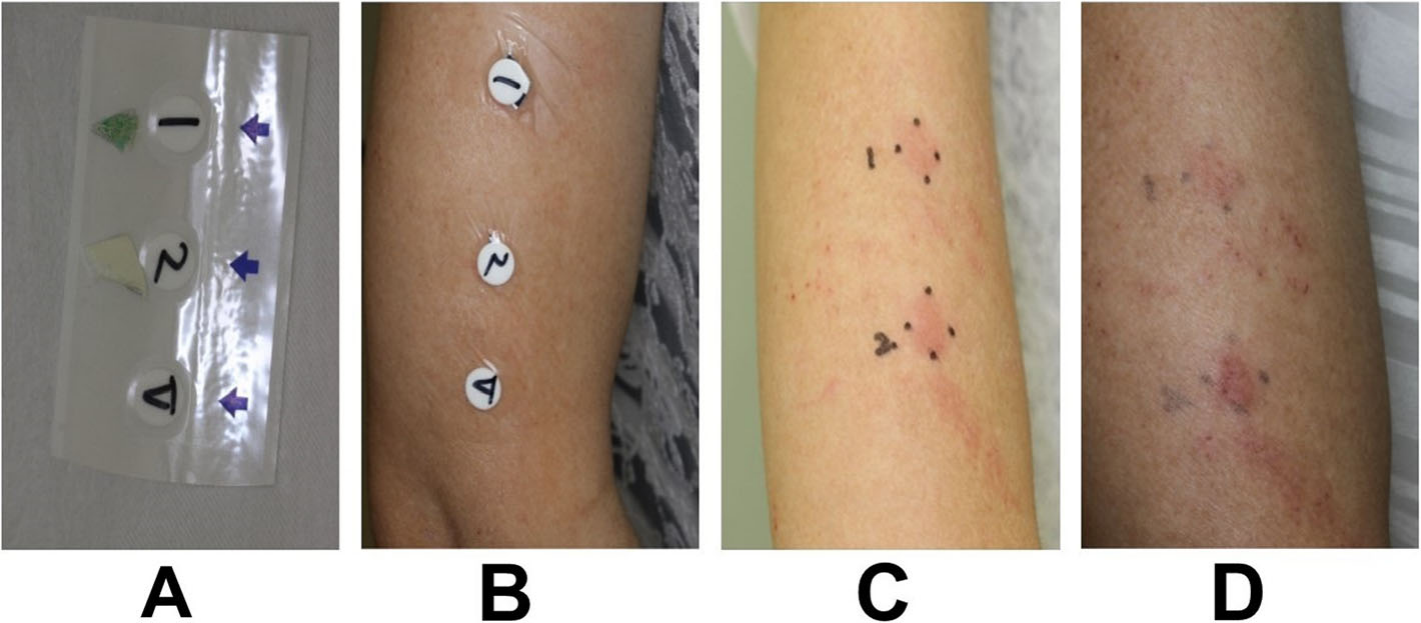
Patch test for the Bispectral Index (BIS) sensor. **a** Patches of each part of the BIS sensor: (1): foam component, (2): adhesive component without Ag electrodes, (V): Vaseline as a control. **b** Attachment site of each component. **c** Positive reactions to the foam component of the BIS sensor (1) and adhesive component without Ag electrodes (2) at 48 h. **d** The positive reaction was maintained at (2) but diminished at (1) at 72 h

One month after surgery, patch testing for allergic reactions to the BIS sensor was performed. After 48 and 72 h, a positive reaction to the adhesive component of the BIS sensor was observed. The reaction to the foam component was positive after 48 h, but it tended to disappear after 72 h (Fig. 2). The patient also had reactions to *p*-tert-butylphenol-formaldehyde resin (PTBP-F-R) and lanolin using a Japanese standard patch test series.

At the outpatient examination 42 days after surgery, erythema had disappeared, and slight pigmentation was observed. In consultation with the patient, the medical treatment was ended at this point.

## Discussion and conclusions

Contact dermatitis associated with anesthesia can be attributable to ECG electrodes [1–9], Prone Positioner™ [10], and contaminants on facemasks [11]; however, the incidence is low, and it often is impossible to sufficiently investigate the causes. We experienced a case of contact dermatitis caused by a BIS sensor, and the results of our patch test identified the adhesive part of the sensor as the cause.

BIS sensors are based on Zipprep™ technology for ensuring good skin contact and optimal signal quality. The Ag-AgCl electrode is printed on polyester material, and acrylic adhesive and foam are attached to it. Previous reports of skin lesions caused by the BIS sensor observed spotted bleeding and erythema on the area in contact with the foam and blisters on the area in contact with the adhesive [12, 13]. In addition, the researchers presumed that these events were caused by the AgCl electrode or an external force such as a pulling force or the prone position [13, 14].

To our knowledge, there have been two reports of contact dermatitis associated with BIS sensors. One patient was in the prone position, and it could not be denied that the event was possibly caused by physical pressure. Both examples were presumed to involve irritant contact dermatitis based on the acute onset on the day of surgery [14, 15]. Patch tests were not conducted in any cases, and no definitive diagnosis was made. From our patch test results, dermatitis in our case was confirmed to be a response to the BIS sensor content. In addition, this report is considered meaningful for examining future cases in which allergy cannot be denied because of the course of onset.

The results of the Japanese standard patch test series performed simultaneously suggested the possibility that PTBP-F-R or lanolin was the causative agent. Although PTBP-F-R is a commonly used ingredient in adhesives and lanolin is sometimes used as a moisturizer or drug base, to our knowledge, no medical accessories have been found that intentionally contained these substances. According to the manufacturer’s description, it is not entirely impossible for these substances to be included in the adhesive component, but it is generally improbable. Patch testing did not help to identify the causative agent in this case. No skin reaction occurred when the ECG electrodes and eye patches were used at the same time. From this phenomenon, it can be inferred that the substance contained on the adhesive side of the electrode differs depending on the product and that any substance contained in the BIS sensor can cause dermatitis.

Avenel-Audran et al. [2] reported similar cases of contact dermatitis caused by ECG electrodes. They could not obtain information regarding the presence of PTBP-F-R in the electrodes from the manufacturers. They performed chemical analysis of electrode samples using gas chromatography–mass spectrometry and high-pressure liquid chromatography and demonstrated the presence of several PTBP-F-R derivatives in the electrodes [2]. We could not perform chemical analysis of the BIS electrodes, but because the manufacturer stated that the possibility of contamination by these substances during manufacturing is not zero, they cannot be dismissed as possible causes (personal communication with the manufacturer). To date, there have been several reports of allergic reactions to ECG electrodes, and the causative substances included PTBP-F-R [2], propylene glycol [4, 5], acrylates [6, 7], para-chloro-meta-xylenol [8], and gum tragacanth [9]. It is not surprising that similar events of dermatitis may occur if these substances are present in the BIS sensor.

There are two types of contact dermatitis: allergic and irritant. It was difficult to determine the type of contact dermatitis in this case because histologic examination was not performed over the course of the condition. Allergic contact dermatitis is a delayed-type reaction. Typically, itching appears 12 h after contact, and it can result in erythematous/edematous changes. Conversely, symptoms of irritant contact dermatitis appear earlier than those of allergic dermatitis. In this case, onset occurred 3 days after surgery, and the erythematous reaction peaked 5 days after contact. Although the patient did not develop any allergic symptoms prior to surgery, the onset on the third postoperative day indicates the possibility that the patient had allergic contact dermatitis.

In recent years, sensors for intraoperative electroencephalography have become larger. Because the sensor is attached on the forehead, dermatitis, which can lead to pigmentation, can result in serious cosmetic implications. It is important to recognize that complications similar to those associated with ECG electrodes can occur with these sensors, even after discharge.

## Data Availability

The relevant data to this case report are unavailable for public access because of patient privacy concerns.

## Abbreviations

BIS: Bispectral Index™
ECG: electrocardiogram
PTBP-F-R: *p*-Tert-butylphenol-formaldehyde resin
